# Hearing Loss Is Associated with Increased Variability in Double Support Period in the Elderly

**DOI:** 10.3390/s21010278

**Published:** 2021-01-04

**Authors:** Betsy Szeto, Damiano Zanotto, Erin M. Lopez, John A. Stafford, John S. Nemer, Adam R. Chambers, Sunil K. Agrawal, Anil K. Lalwani

**Affiliations:** 1 Division of Otology, Neurotology, and Skull Base Surgery, Department of Otolaryngology—Head and Neck Surgery, Columbia University Vagelos College of Physicians and Surgeons, New York, NY 10032, USA; bs2898@cumc.columbia.edu (B.S.); erin.mamuyac@unchealth.unc.edu (E.M.L.); staffojh@ucmail.uc.edu (J.A.S.); jsn2130@columbia.edu (J.S.N.); adam.rouman@gmail.com (A.R.C.); 2 Department of Mechanical Engineering, Stevens Institute of Technology, School of Engineering & Science, Hoboken, NJ 07030, USA; dzanotto@stevens.edu; 3 Department of Mechanical Engineering, School of Engineering and Applied Science, Columbia University, New York, NY 10027, USA; sa3077@columbia.edu; 4 Department of Rehabilitative and Regenerative Medicine, Columbia University Medical Center, New York, NY 10032, USA

**Keywords:** spatiotemporal gait parameters, hearing loss, ambulatory gait analysis, wearable technology, instrumented footwear

## Abstract

Hearing loss is a disabling condition that increases with age and has been linked to difficulties in walking and increased risk of falls. The purpose of this study is to investigate changes in gait parameters associated with hearing loss in a group of older adults aged 60 or greater. Custom-engineered footwear was used to collect spatiotemporal gait data in an outpatient clinical setting. Multivariable linear regression was used to determine the relationship between spatiotemporal gait parameters and high and low frequency hearing thresholds of the poorer hearing ear, the left ear, and the right ear, respectively, adjusting for age, sex, race/ethnicity, and the Dizziness Handicap Inventory–Screening version score. Worsening high and low frequency hearing thresholds were associated with increased variability in double support period. Effects persisted after adjusting for the effects of age and perceived vestibular disability and were greater for increases in hearing thresholds for the right ear compared to the left ear. These findings illustrate the importance of auditory feedback for balance and coordination and may suggest a right ear advantage for the influence of auditory feedback on gait.

## 1. Introduction

Hearing loss is a disabling condition that increases with age and affects nearly two-thirds of adults aged 70 and over in the US population [1]. Among older adults, hearing loss has been linked to difficulties in walking and increased risk of falls [2,3], and some studies have suggested that the use of hearing aids may reduce the risk of falls in this group [4]. A recent retrospective cohort analysis of adults over the age of 18 in an inpatient setting showed that patients with hearing loss who did not have hearing aids had an increased risk of falls, even after controlling for age and sex; this effect was not present in patients with hearing loss who had hearing aids [5]. While there is a growing body of evidence that untreated hearing loss may lead to increased risk of falls, the mediating factors of this relationship remain unelucidated.

Falls are increasingly common with age, with more than a quarter of adults over the age of 65 falling at least once a year in the US [6,7]. Falls are tremendously detrimental to the quality of life of older adults and can lead to fear of falls, decreased mobility, injuries, fractures, and mortality [6,7,8,9,10]. Abnormalities in balance and gait are among recognized intrinsic risk factors for falls and are also prevalent in the elderly [11,12]. Indeed, the gait patterns of older adults are characterized by reduced velocity, shorter step length, and increased step timing variability [13], and shorter stride and step length, wider step width, and increased variability in gait parameters have been implicated in increased risk of falls [14]. Perceived vestibular impairment, as determined by the Dizziness Handicap Inventory–Screening version (DHI-S) score, has been found to be associated with impaired gait function [15]. In particular, patients with higher DHI-S score took shorter steps and fewer steps per minute, resulting in slower walking speed, and showed larger variability in temporal gait parameters including cadence, double support period, swing period, and stance-to-swing. It has been suggested, however, that hearing impairment may have effects on postural instability and impaired balance beyond the effects of vestibular dysfunction [16]. Auditory feedback may serve as reafferent signals that are important for locomotion and coordination [17]. While investigating whether step sounds generated during running have impacts on performance of a hurdling task, Kennel et al. found that delayed auditory feedback resulted in slower overall time and altered kinematic parameters [17]. Sallard et al. found age-related declines in processing of sensory reafference as demonstrated by an increased inter-tap interval variability in a bimanual task in the elderly group compared to the younger group [18]. In a recent study, older adults using ear plugs to inhibit auditory feedback were shown to exhibit increases in step length, thought to be a compensation that increased ground reaction forces, allowing participants to sense footsteps [19]. Auditory feedback is thought to regulate gait through providing temporal and spatial information that becomes increasingly valuable with age, as balance deteriorates. Thus, we hypothesized that worsening hearing function in the elderly would be reflected in increased gait variability. To test this hypothesis, we analyzed the gait of a group of older adults aged 60 or greater, as they completed an overground walking task at comfortable speed. We then investigated associations between participants’ spatiotemporal gait parameters and objective measures of their hearing sensitivity.

## 2. Materials and Methods

### 2.1. Participants

Participants were recruited during their visit to the Otology and Neurology clinic at Columbia University, and the study was approved by the local Institutional Review Board. Because the prevalence of hearing loss starts increasing dramatically from the age of 60 onwards [20], only individuals aged 60 or greater were involved in the study. Participants with stroke, Parkinson’s disease, or other medical conditions known to affect gait and participants who had undergone hip or knee surgery were excluded from the study. Eighty participants with available gait data and audiometric data were included for analysis.

### 2.2. Audiometric Data

Audiometric measurements were conducted in a sound-treated double-walled audiometric suite using insert earphones and an audiometer calibrated to the standards set by the American National Standards Institute (ANSI S3.6-2010). Hearing sensitivity was measured through pure tone air conduction audiometry by presenting pure tone signals to the ear through earphones and varying the signal intensity until the hearing threshold was determined for each frequency. Therefore, higher thresholds indicate worse hearing. Pure tone air conduction thresholds at 500, 1000, 2000, 4000, 6000, and 8000 Hz were recorded in decibel hearing level (dB). Pure tone averages (PTA) were determined as the average hearing thresholds in dB at select tested frequencies, with higher PTA representing worse hearing at those frequencies. In this study, low frequency PTA were taken for 500, 1000, and 2000 Hz, and high frequency PTA were taken for 4000, 6000, and 8000 Hz [21]. Low frequency PTA represent frequencies most commonly used in speech, while high frequency PTA represent frequencies preferentially lost in presbycusis. Low frequency PTA and high frequency PTA were calculated for the poorer hearing ear, the right ear, and the left ear. The poorer hearing ear was chosen as it best reflects a potential underlying worsening vestibular system. Additionally, as a right ear advantage has been reported previously for speech [22], left and right ear thresholds were chosen to explore whether associations with gait are stronger on either side.

### 2.3. Instrumented Footwear for Gait Analysis

**Figure 1:**
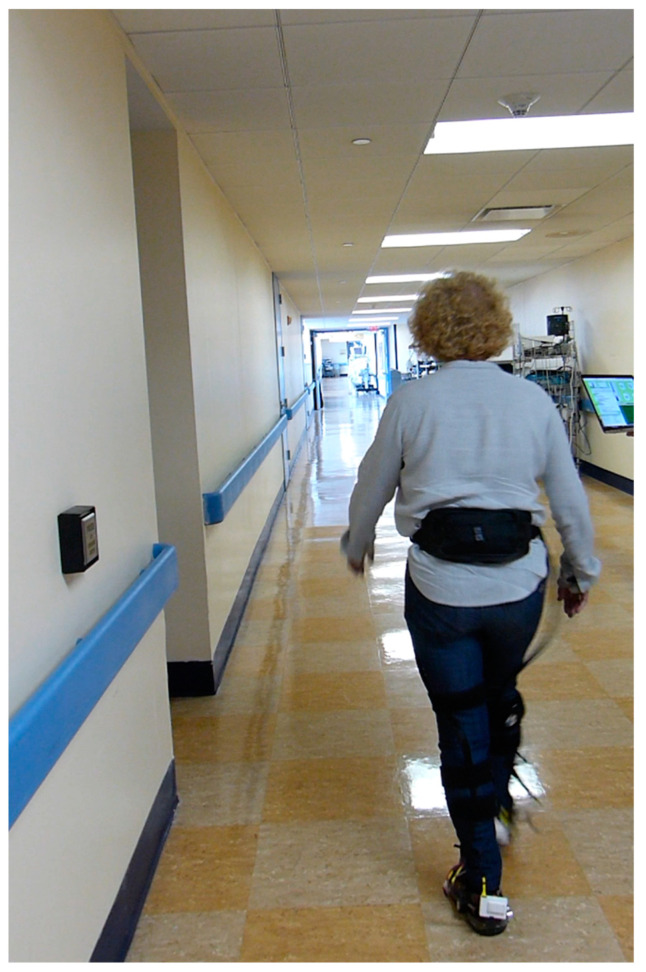
Participant walking while wearing custom-engineered footwear developed in the Columbia University Robotics and Rehabilitation Laboratory. Each participant completed a 100-m-long course.

To collect spatiotemporal gait data in out-of-the-lab conditions, participants were asked to put on custom-engineered footwear developed in the Columbia University Robotics and Rehabilitation Laboratory (Figure 1, [23]). This wearable system is capable of measuring kinematic gait parameters and optionally delivering real-time auditory and vibrotactile feedback in response to those parameters. The gait analysis capability of the instrumented footwear was previously validated with healthy individuals [24] and patients with a neuromuscular disorder affecting the gait function [25]. The system consists of two footwear units and a hip pack unit. Each footwear unit includes four force-sensitive resistors (used as foot switches), an inertial measurement unit (IMU), and five vibrotactile transducers, all embedded in the sole of regular sandals. A second IMU is encased in a small plastic box secured with a Velcro strap to the user’s proximal shank, enabling the system to measure the shank’s kinematic data. An ultrasonic sensor is mounted on the posteromedial side of the sole to estimate stride width. The hip pack unit includes a portable single-board computer, an external sound card used to control the auditory and vibrotactile feedback, a small Wi-Fi router, and a Li-Po battery. The single-board computer synchronizes the data incoming from the footwear units, runs the feedback engine, and performs data-logging to a micro-SD card at a sample rate of 500 Hz. The total weight of the hip pack unit is 1.14 kg, and the weight of the components attached to each sandal is 0.19 kg. In this study, the feedback capability of the device was not used.

### 2.4. Experimental Protocol for Measurement of Gait Parameters

Participants chose an appropriate shoe size for the instrumented footwear. Subsequently, wearing the device, each participant completed four uninterrupted walking laps along a 25-m-long straight-line path, covering a total of 100 m at their chosen pace (Figure 1).

Gait parameters analyzed include stride length, cadence, walking speed, foot-ground clearance, swing period, double-support time, and stance-to-swing (i.e., the intra-limb ratio between the duration of the stance phase and that of the swing phase within one stride). For stride length, stride height, and stride velocity, normalized metrics adjusting for the subject’s stature (as described in [26]) were also analyzed. For each of these gait parameters, we extracted 40 consecutive left and right strides of steady-state walking within each lap, resulting in 160 samples for each parameter, for each participant. These samples were subsequently used to compute the mean and coefficient of variation (CV) for each individual. This large number of samples was chosen to provide a reliable estimate of gait variability [27]. Additionally, the confounding effects of gait asymmetries on gait variability were compensated for by calculating the standard deviation from residuals of each stride around the mean over the corresponding limb [28]. This method also allows for a more precise measure of gait variability, as it doubles the number of samples that can be used in the analysis.

### 2.5. Other Variables

Other variables of interest collected include age, sex, race/ethnicity, and the DHI-S score. The DHI-S is an abbreviated 10-question version of the original 25-question DHI score, which is used the quantify perceived vestibular disability [29,30]. The DHI-S score has been shown to be highly correlated with the original DHI [31]. Participants completed the DHI-S survey prior to measurement of gait parameters, and the DHI-S score was tabulated from the responses.

### 2.6. Statistical Analysis

Multivariable linear regression was employed to determine whether high and low frequency PTA of the poorer hearing ear, the left ear, and the right ear were associated with changes in gait parameters, adjusting for age, sex, race/ethnicity, and DHI-S score, which are known to affect gait patterns [15]. Models including PTA of the left or right ear were additionally adjusted for the laterality of the poorer hearing ear (i.e., right or left ear). Given the limited sample size, race/ethnicity was categorized into two groups: non-Hispanic White and Other. This categorization resulted in an even distribution of subjects across both groups. Separate linear regression models were fit to each gait parameter. Standardized coefficients were used to assess the relative importance of independent variables.

The assumption of normal distribution of the residuals was checked by inspecting the normal probability plots of the standardized residuals. Deviations from linearity and homoscedasticity were identified by inspecting the scatterplots of the standardized residuals plotted against the standardized predicted values. We checked for potential multicollinearity among predictors using tolerance and reciprocal of the variance inflation factor, with a threshold of 0.2. The assumption of independent errors was checked using the Durbin–Watson statistic.

Influential outliers were identified using the following criteria: 1) absolute value of the externally studentized residual > critical t, where df = N − p −1 with N, p being the # of data points and the # of predictors, respectively [32], and 2) either Cook’s D>1 [33] or leverage > 2p/N [34]. Sensitivity analyses were conducted including and excluding these identified outliers. All analyses were conducted using SAS 9.4 (SAS Institute, Cary, NC, USA).

## 3. Results

### 3.1. Characteristics

The characteristics of the study sample are summarized in Table 1. The study sample included 80 participants, aged 60 to 95 (mean 73.7, SD 8.8). A total of 46.2% of the sample was female and 52.5% were Non-Hispanic White; 87.5% of participants had hearing loss as a chief complaint, 42.5% had tinnitus, and 51.3% had dizziness or imbalance. Low frequency hearing thresholds were similar between the right (37.4 ± 23.9 dB) and left (37.4 ± 23.7 dB) ears. High frequency hearing thresholds were higher for both the right (52.2 ± 23.5 dB) and left (55.9 ± 24.5 dB) ears.

The mean and CV of all gait parameters are summarized in Table 2. Because of technical problems with one of the instrumented sandals, bilateral metrics were not available for 6 participants, resulting in 74 valid data points.

### 3.2. Multiple Linear Regression

To investigate the relationship between hearing thresholds and gait parameters, linear regression models were employed to model the relationship between each gait parameter as an outcome and the high frequency and low frequency PTA of the poorer hearing ear, respectively, adjusting for age, sex, race/ethnicity, and DHI-S score. Outliers were not excluded from further analyses as there was no evidence of physiologically implausible values indicating measurement errors. A 10 dB increase in high frequency PTA in the poorer hearing ear was associated with an increase in double support period (DSP) CV by 0.814 percentage points (*p* < 0.01), a decrease in mean normalized stride height by 0.130 percentage points (*p* < 0.05), and no significant changes in other gait parameters (Table S1). A 10 dB increase in low frequency PTA in the poorer hearing ear was associated with an increase in DSP CV by 0.671 percentage points (*p* = 0.01) and no changes in other gait parameters (Table S2).

For DSP CV and stride height, linear regression models were also used to model the relationship between these gait parameters and high and low frequency hearing thresholds of the right and left ear. These models were adjusted for age, sex, race/ethnicity, DHI-S score, and the laterality of the poorer hearing ear.

**Figure 2:**
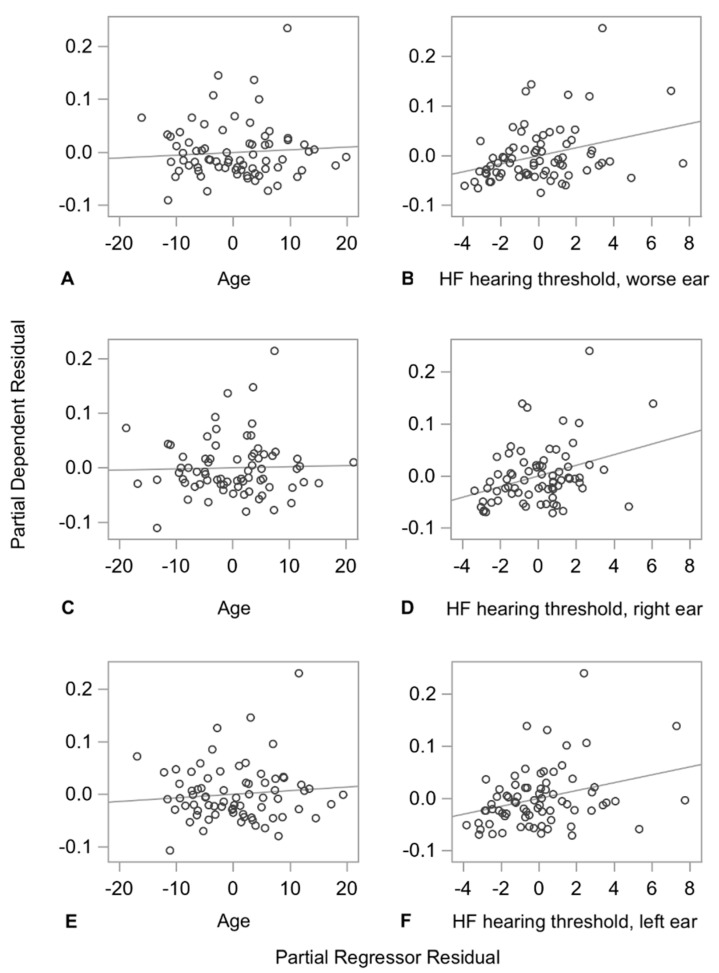
Partial regression plots for double support period (DSP) CV for age, and high frequency hearing thresholds for the poorer hearing ear, the right ear, and the left ear. Each plot illustrates the strength of the relationship between DSP CV and age (**A**,**C**,**E**), or high frequency hearing thresholds of the poorer hearing ear (**B**), the right ear (**D**), or the left ear (**F**). The y axis represents the residuals from regressing DSP CV against all the predictors but one (age, or high frequency hearing threshold). The x axis represents the residuals from regressing the omitted predictor against the remaining predictors in the model. There is a strong positive relationship between variability in DSP and high frequency hearing thresholds for the poorer hearing ear, the right ear, and the left ear, respectively. HF indicates high frequency.

The DSP CV (Table 3) increased by 1.022 (*p* < 0.01) and 0.759 (*p* < 0.01) percentage points with every 10 dB increase in high frequency hearing thresholds in the right ear and the left ear, respectively; this increase was greater for increases in the right ear high frequency PTA. All standardized coefficients for the high frequency hearing thresholds were greater than the standardized coefficients for age in the same model, suggesting that the predictive ability of high frequency hearing loss for DSP CV is stronger than that of age. Figure 2 shows the partial regression plots for DSP CV for age and high frequency hearing thresholds for the poorer hearing ear, the right ear, and the left ear.

**Figure 3:**
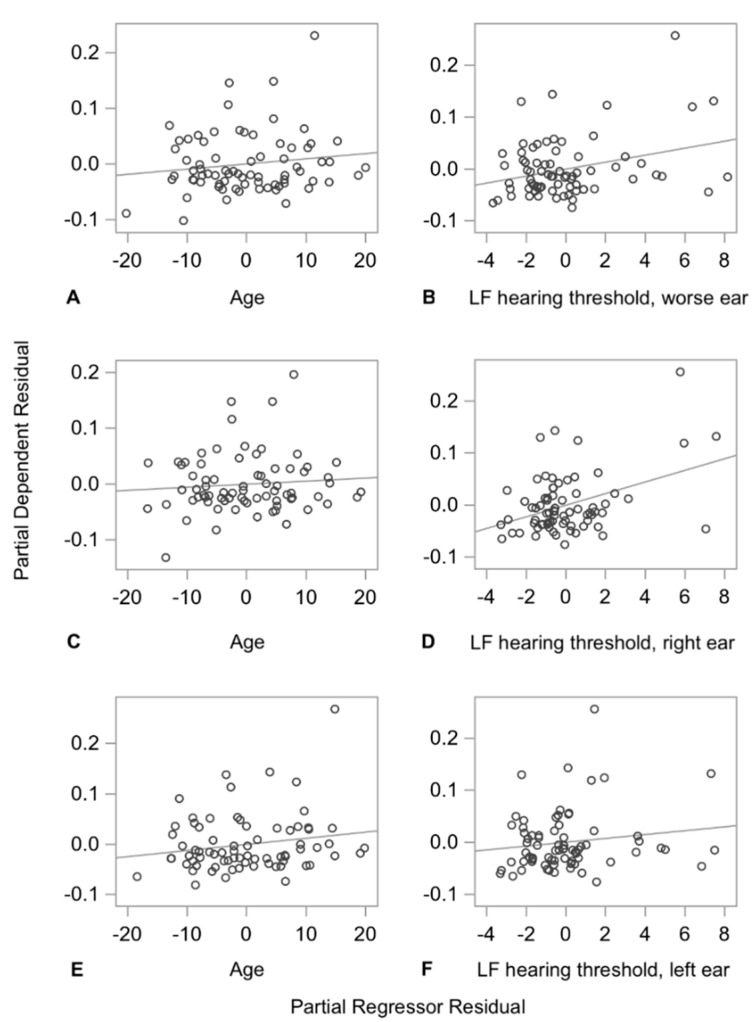
Partial regression plots for double support period (DSP) CV for age and low frequency hearing thresholds for the poorer hearing ear, the right ear, and the left ear. Each plot illustrates the strength of the relationship between DSP CV and age (**A**,**C**,**E**), or low frequency hearing thresholds of the poorer hearing ear (**B**), the right ear (**D**), or the left ear (**F**). There is a strong positive relationship between variability in DSP and low frequency hearing thresholds for the poorer hearing ear and the right ear, respectively. LF indicates low frequency.

The DSP CV increased by 1.114 (*p* = 0.0006) percentage points with every 10 dB increase in low frequency hearing thresholds in the right ear, but not the left ear. The standardized coefficients for the low frequency hearing thresholds of the poorer hearing ear and the right ear were greater than the standardized coefficients for age in the same model, suggesting that the predictive ability of low frequency hearing loss for DSP CV is stronger than that of age. Figure 3 shows the partial regression plots for DSP CV for age and low frequency hearing thresholds for the poorer hearing ear, the right ear, and the left ear.

Stride height was only associated with high frequency hearing thresholds in the poorer hearing ear (Table 4).

## 4. Discussion

This study examined the relationship between spatiotemporal gait parameters and hearing thresholds in a group of older adults aged 60 to 95. The variability in DSP was found to increase with worsening high and low frequency hearing thresholds. The effect of hearing thresholds was greater than the effect of age on this gait parameter, as demonstrated by the magnitude of the standardized coefficients. The increase in DSP variability was greater for increases in hearing thresholds for the right ear compared to the left ear. Stride height was also found to decrease with increases in the high frequency hearing thresholds of the poorer hearing ear. No association was found between hearing thresholds and all other gait parameters included in the analysis.

Studies investigating the relationship between age, falls, and gait parameters have identified increased variability in gait parameters as a risk factor for falls that increases with age [13,35,36]. Furthermore, gait variability is a hallmark of fear of falling [37]. In particular, increased variability in the DSP has been linked to increased risk of multiple falls in older adults [38]. In nursing home residents with dementia, an increase in double support time variability by 10 percentage points was found to be associated with an increase in odds of falling within a 3-month period by 53% [39]. Double support time variability has also been linked to vestibular asymmetry [40,41], with variability ranging from 2.38% to 3.0% larger in individuals with vestibular asymmetries [41]. DSP is the only interlimb gait parameter analyzed in this study. It defines the period of time during which both feet are in contact with the ground, as the swinging leg meets the ground, and weight is transferred from the support leg to the swinging leg [42]. Increased variability in this gait parameter may indicate poor interlimb coordination and deteriorated balance-control mechanisms [43]. Interlimb coordination has been found to decline with age [44], and impaired interlimb coordination is associated with increased risk of developing mobility limitations [45]. Auditory feedback may be important for coordination by providing temporal and spatial information. In a recent study, Stepanchenko et al. found that deaf children exhibited poorer coordination of the hands and feet compared to healthy controls [46]. Auditory feedback has been found to be important for integrated timing of both hands in the learning of a bimanual task [47] and in improving interlimb coordination in juggling [48]. In this study, we found that variability in DSP increased with increasing hearing thresholds, even after adjusting for age, suggesting that auditory feedback may be important for coordination of both legs during locomotion. It is possible that increased variability in this gait parameter may be one of the mediating factors explaining the relationship between hearing loss and falls.

In a previous study, perceived vestibular impairment, as determined by increased DHI-S score, was associated with changes in several gait parameters, including reduced stride length, cadence, and walking speed, and increased variability in cadence, DSP, swing period, and stance-to-swing [15]. As the auditory and peripheral vestibular systems are intricately linked, one may expect the function of the auditory system to mirror that of the vestibular system, thus hypothesizing that hearing loss may have effects on gait parameters similar to those of vestibular impairment. In contrast, it has been suggested that hearing impairment may have effects on postural instability and impaired balance even beyond the effects of vestibular dysfunction [16]. Of note, the association of increased variability in DSP with increased hearing thresholds persisted even after adjusting for DHI-S score, suggesting that auditory feedback is important for gait coordination such that hearing loss has detrimental effects beyond the effect of vestibular impairment.

Another interesting finding in this study is that the variability of DSP increases more with hearing threshold increases in the right ear compared to the left ear. A right ear advantage for speech has been well-established, and it is thought to reflect left-hemispheric dominance [22]. Right-ear dominance has been demonstrated using otoacoustic emissions and auditory brainstem responses [49], and in children receiving bilateral cochlear implants [50]. Further investigation should determine whether a right ear advantage may also manifest for auditory feedback influencing gait.

This study has several strengths. While hearing loss increases with age, and has also been linked to falls, few studies have investigated the linkage between hearing loss and gait parameters. The use of a custom-engineered footwear-based gait analysis system enabled the collection of spatiotemporal gait parameters with high granularity, in the clinical setting, and without the space constraints associated with traditional gait laboratory equipment. The availability of rich data on hearing thresholds and spatiotemporal gait parameters enabled a thorough analysis of the relationship between spatiotemporal gait parameters and high and low frequency hearing thresholds in the right ear, left ear, and poorer hearing ear.

Our study is limited by its small sample size, which may have affected the ability to detect relationships. Furthermore, it also limited the number of variables we could adjust for in our multivariable analyses; however, we adjusted for age, sex, race, and DHI-S score, which have been previously linked to changes in gait parameters. Prospective falls were not included in this analysis; therefore, definitive conclusions about the role of DSP variability as a mediator between hearing loss and falls cannot be made. Further investigation is warranted to understand these relationships. Finally, ongoing efforts are directed toward improving form factor, weight, accuracy, and usability of the footwear-based gait analysis system [51].

## 5. Conclusions

In older adults, worsening high and low frequency hearing thresholds were associated with increased variability in DSP. Effects persisted after adjusting for the effects of age and perceived vestibular disability, suggesting that auditory feedback may be important for balance control and interlimb coordination beyond the effects of the peripheral vestibular system. Additionally, the increase in DSP variability was greater for increases in hearing thresholds for the right ear compared to the left ear. Future studies may investigate whether there is a right ear advantage for the influence of auditory feedback on gait.

## Figures and Tables

**Table 1:** Demographic characteristics of study sample.

Characteristic	N = 80
Age, mean ± SD	73.7 ± 8.8
Sex, N (%)	
Male	37 (46.2)
Female	43 (53.8)
Race/Ethnicity, N (%)	
Non-Hispanic White	42 (52.5)
All others	38 (47.5)
BMI, kg/m^2^, mean ± SD	26.3 ± 4.0
Height, m, mean ± SD	1.67 ± 0.09
Weight, kg, mean ± SD	71.2 ± 15.2
Hearing parameters, mean ± SD	
Low frequency PTA, poorer hearing ear	44.4 ± 27.2
Low frequency PTA, right ear	37.4 ± 23.9
Low frequency PTA, left ear	37.4 ± 23.7
High frequency PTA, poorer hearing ear	61.1 ± 24.7
High frequency PTA, right ear	52.2 ± 23.5
High frequency PTA, left ear	55.9 ± 24.5
Low frequency PTA, poorer hearing ear, N (%)	
0 to 25 dB	22 (27.5)
>25 dB to 40 dB	22 (27.5)
>40 dB to 60 dB	22 (27.5)
>60 dB to 80 dB	6 (7.5)
>80 dB	8 (10.0)
High frequency PTA, poorer hearing ear, N (%)	
0 to 25 dB	4 (5.0)
>25 dB to 40 dB	14 (17.5)
>40 dB to 60 dB	23 (28.8)
>60 dB to 80 dB	21 (26.3)
>80 dB	18 (22.5)
DHI-S score, mean ± SD	6.3 ± 8.5
Chief complaints	
Hearing loss	70 (87.5)
Tinnitus	34 (42.5)
Dizziness or Imbalance	41 (51.3)
Other	12 (15.2)

Abbreviations: PTA, pure tone average; DHI-S, Dizziness Handicap Inventory–Screening version score.

**Table 2:** Mean and coefficient of variation (CV) for temporal and spatial gait parameters.

Gait Parameter	N	Mean	SD	Min	Max
Cadence					
Mean (stp/min)	80	108	10.7	73.6	130.6
CV (%)	80	2.7	1	1.1	6.1
Double supp. period					
Mean (%)	74	10	2.6	4.8	15.5
CV (%)	74	10.4	6	4.1	40.9
Stride Height					
Mean (m)	80	0.15	0.026	0.091	0.257
CV (%)	80	9.5	9.9	2.5	45.5
Normalized Stride Height					
Mean (%)	80	9.1	1.4	5.6	14.5
CV (%)	80	9.5	9.9	2.5	45.5
Stride Length					
Mean (m)	80	1.223	0.21	0.583	1.592
CV (%)	80	4.8	2.8	1.9	16.6
Normalized Stride Length					
Mean (%)	80	73.7	11.7	35.9	93.6
CV (%)	80	4.8	2.8	1.9	16.6
Stance-to-swing					
Mean	80	1.52	0.16	1.22	1.9
CV (%)	80	5.7	3.3	2.3	22.6
Swing period					
Mean (%)	80	39.9	2.5	34.5	45.2
CV (%)	80	3.3	1.6	1.3	9.9
Walking Speed					
Mean (m/s)	80	1.111	0.243	0.475	1.54
CV (%)	80	5.9	3.3	2.4	20.6
Normalized Walking Speed					
Mean (%)	80	27.5	5.9	11.9	37.7
CV (%)	80	5.9	3.3	2.4	20.6

Abbreviations: CV, coefficient of variation.

**Table 3:** Multiple regression models for the outcome double support period CV for 10 dB increases in different hearing thresholds.

Outcome	PTA	N	R^2^	B_PTA_	*p*-Value	B_DHI-S_	*p*-Value	B_age_	*p*-Value	β_PTA_	β_DHI-S_	β_age_
Double supp. period CV (%)	HF, poorer ear	74	0.1895	0.814	**0.0055**	0.092	0.2300	0.051	0.5286	0.33449	0.13381	0.07512
	HF, right ear	74	0.2277	1.022	**0.0042**	0.094	0.2143	0.020	0.8186	0.38664	0.13720	0.02889
	HF, left ear	74	0.2099	0.759	**0.0096**	0.105	0.1728	0.068	0.4011	0.31613	0.15209	0.10006
	LF, poorer ear	74	0.1750	0.671	**0.0107**	0.053	0.5055	0.092	0.2400	0.30466	0.07677	0.13561
	LF, right ear	74	0.2397	1.114	**0.0006**	0.043	0.5842	0.054	0.4819	0.43071	0.06183	0.07994
	LF, left ear	74	0.1118	0.372	0.2209	0.090	0.2792	0.121	0.1347	0.15138	0.13063	0.17860

Abbreviations: PTA, pure tone average; HF, high frequency; LF, low frequency; DHI-S, Dizziness Handicap Inventory–Screening version score; CV, coefficient of variation; R^2^, coefficient of determination; B, unstandardized regression coefficient; β, standardized regression coefficient. *p*-Values < 0.05 are bolded.

**Table 4:** Multiple regression models for the outcome normalized stride height (%) for 10 dB increases in different hearing thresholds.

Outcome	PTA	N	R^2^	B_PTA_	*p*-Value	B_DHI-S_	*p*-Value	B_age_	*p*-Value	β_PTA_	β_DHI-S_	β_age_
Normalized stride height, mean (%)	HF, poorer ear	80	0.1535	−0.130	**0.0491**	0.007	0.7059	−0.019	0.3052	−0.22689	0.04105	−0.11807
	HF, right ear	80	0.1514	−0.136	0.0859	0.009	0.6160	−0.016	0.4159	−0.22588	0.05556	−0.09887
	HF, left ear	80	0.1588	−0.129	0.0581	0.009	0.6027	−0.018	0.3391	−0.22517	0.05744	−0.11150
	LF, poorer ear	80	0.1293	−0.079	0.1794	0.011	0.5625	−0.027	0.1353	−0.15257	0.06531	−0.16786
	LF, right ear	80	0.1414	−0.081	0.2555	0.015	0.4326	−0.027	0.1438	−0.13759	0.09062	−0.16633
	LF, left ear	80	0.1401	−0.075	0.2771	0.014	0.4673	−0.029	0.1041	−0.12682	0.08328	−0.18171

Abbreviations: PTA, pure tone average; HF, high frequency; LF, low frequency; DHI-S, Dizziness Handicap Inventory–Screening version score; CV, coefficient of variation; R^2^, coefficient of determination; B, unstandardized regression coefficient; β, standardized regression coefficient. *p*-Values < 0.05 are bolded.

## Data Availability

The data presented in this study are available on request from the corresponding author. The data are not publicly available due to participant confidentiality.

## Supplementary Materials

The following are available online at https://www.mdpi.com/1424-8220/21/1/278/s1, Table S1: Multiple regression models for 10 dB increases in high frequency pure tone averages of the poorer hearing ear, Table S2: Multiple regression models for 10 dB increases in low frequency pure tone averages of the poorer hearing ear.
