# Supplementary material for: Hearing Loss Is Associated with Increased Variability in Double Support Period in the Elderly
Source: Sensors (Basel). 2021 Jan 4;21(1):278. doi: 10.3390/s21010278 (PMC7795333; doi:10.3390/s21010278)
Supplement: Supplementary file 1 [file sensors-21-00278-s001.pdf]

Supplementary material for "Hearing loss is associated with increased variability in double support period in the elderly", Szeto et al.

Supplementary Table 1: Multiple regression models for 10 dB increases in high frequency pure tone averages of the poorer hearing ear

Gait parameter	N	R-squared	B_PTA	p-value	B_DHI	p-value	B_age	p-value	B_male	p-value	B_nhwhite	p-value
Cadence												
Mean (steps/min)	80	0.1391	0.35639	0.4732	-0.22942	0.0974	-0.36935	0.0099	-3.70763	0.1117	2.29193	0.3230
CV (%)	80	0.3274	-0.001437	0.9731	0.028317	0.0184	0.059394	<.0001	-0.150	0.4525	-0.368	0.0672
Double supp. period												
Mean (%)	74	0.0583	-0.163	0.2099	0.035129	0.3125	0.061086	0.0998	0.178	0.7641	0.337	0.5770
CV (%)	74	0.1895	0.814	**0.0055**	0.092120	0.2300	0.051082	0.5286	-0.757	0.5636	-2.188	0.1033
Stride Height												
Mean (m)	80	0.2938	-0.00224	**0.0401**	0.00004049	0.8919	-0.00045372	0.1390	0.02301	<.0001	0.00251	0.6171
CV (%)	80	0.0966	-0.607	0.2002	0.143	0.2756	0.245	0.0682	1.724	0.4333	-3.603	0.1038
Normalized Stride Height												
Mean (%)	80	0.1535	-0.130	**0.0491**	0.006784	0.7059	-0.018887	0.3052	0.765	0.0134	0.030245	0.9205
CV (%)	80	0.0966	-0.607	0.2002	0.143	0.2756	0.245	0.0682	1.724	0.4333	-3.603	0.1038
Stride Length												
Mean (m)	80	0.2848	-0.00379	0.6704	-0.00363	0.1432	-0.00963	0.0003	0.11217	0.0083	0.05094	0.2220
CV (%)	80	0.1573	-0.031224	0.8109	0.048351	0.1827	0.106	0.0051	-0.962	0.1165	-0.463	0.4476
Normalized Stride Length												
Mean (%)	80	0.1997	-0.147	0.7794	-0.189	0.1953	-0.539	0.0005	1.515	0.5365	2.388	0.3307
CV (%)	80	0.1573	-0.031224	0.8109	0.048351	0.1827	0.106	0.0051	-0.962	0.1165	-0.463	0.4476
Swing period												
Mean (%)	80	0.0311	0.088899	0.4700	-0.034221	0.3153	-0.042026	0.2280	-0.07415	0.8969	-0.074950	0.8958
CV (%)	80	0.1991	0.050179	0.4866	0.035506	0.0776	0.063522	0.0025	0.253	0.4525	-0.527	0.1192
Stance-to-swing												
Mean	80	0.0358	-0.00507	0.5232	0.00253	0.2501	0.00287	0.2020	0.00841	0.8200	0.00140	0.9698
CV (%)	80	0.1611	0.188	0.2203	0.085626	0.0449	0.087770	0.0442	0.146	0.8367	-1.150	0.1083
Walking Speed												
Mean (m/s)	80	0.2539	-0.00117	0.9120	-0.00529	0.0726	-0.01212	0.0001	0.06351	0.1986	0.06075	0.2186
CV (%)	80	0.1905	-0.064270	0.6663	0.078453	0.0598	0.135	0.0020	-1.052	0.1326	-0.706	0.3110
Normalized Walking Speed												
Mean (%)	80	0.2279	-0.015648	0.9522	-0.126	0.0834	-0.293	0.0002	0.584	0.6310	1.382	0.2577
CV (%)	80	0.1905	-0.064270	0.6663	0.078453	0.0598	0.135	0.0020	-1.052	0.1326	-0.706	0.3110

Abbreviations: PTA, pure tone average; DHI-S, Dizziness Handicap Inventory – Screening version score; nhwhite, non-Hispanic white; CV, coefficient of variation; R^2, coefficient of determination; B, unstandardized regression coefficient

Supplementary material for "Hearing loss is associated with increased variability in double support period in the elderly", Szeto et al.

Supplementary Table 2: Multiple regression models for 10 dB increases in low frequency pure tone averages of the poorer hearing ear

Gait parameter	N	R^2	B_{PTA}	p-value	B_{DHI-S}	p-value	B_{age}	p-value	B_{male}	p-value	$B_{nhwhite}$	p-value
Cadence												
Mean (steps/min)	80	0.1360	0.21956	0.6190	-0.24059	0.0896	-0.34756	0.0114	-3.49204	0.1350	2.30674	0.3207
CV (%)	80	0.3318	-0.026086	0.4900	0.030026	0.0143	0.060587	<.0001	-0.162	0.4150	-0.369	0.0658
Double supp. period												
Mean (%)	74	0.0587	-0.147	0.2053	0.043927	0.2203	0.053583	0.1303	0.032445	0.9567	0.330	0.5845
CV (%)	74	0.1750	0.671	**0.0107**	0.052853	0.5055	0.092217	0.2400	-0.070926	0.9575	-2.144	0.1133
Stride Height												
Mean (m)	80	0.2744	-0.00146	0.1363	0.00011568	0.7079	-0.00058723	0.0500	0.02162	<.0001	0.00242	0.6351
CV (%)	80	0.1119	-0.712	0.0889	0.184	0.1660	0.225	0.0776	1.200	0.5823	-3.638	0.0977
Normalized Stride Height												
Mean (%)	80	0.1293	-0.079048	0.1794	0.010794	0.5625	-0.026852	0.1353	0.687	0.0281	0.024883	0.9354
CV (%)	80	0.1119	-0.712	0.0889	0.184	0.1660	0.225	0.0776	1.200	0.5823	-3.638	0.0977
Stride Length												
Mean (m)	80	0.2834	-0.00157	0.8427	-0.00356	0.1599	-0.00991	<.0001	0.11023	0.0096	0.05081	0.2236
CV (%)	80	0.1621	-0.080161	0.4884	0.053352	0.1494	0.107	0.0031	-1.009	0.0995	-0.466	0.4432
Normalized Stride Length												
Mean (%)	80	0.1989	0.006775	0.9884	-0.191	0.2008	-0.553	0.0002	1.471	0.5492	2.385	0.3316
CV (%)	80	0.1621	-0.080161	0.4884	0.053352	0.1494	0.107	0.0031	-1.009	0.0995	-0.466	0.4432
Swing period												
Mean (%)	80	0.0330	0.089229	0.4138	-0.039284	0.2591	-0.038342	0.2504	-0.004473	0.9938	-0.070242	0.9022
CV (%)	80	0.1955	-0.025471	0.6911	0.037656	0.0678	0.069454	0.0006	0.257	0.4465	-0.527	0.1203
Stance-to-swing												
Mean	80	0.0383	-0.00545	0.4394	0.00284	0.2059	0.00268	0.2133	0.00427	0.9080	0.00112	0.9758
CV (%)	80	0.1442	-0.026875	0.8440	0.089151	0.0431	0.106	0.0123	0.194	0.7869	-1.146	0.1129
Walking Speed												
Mean (m/s)	80	0.2538	-0.00067368	0.9426	-0.00526	0.0806	-0.01220	<.0001	0.06283	0.2041	0.06070	0.2190
CV (%)	80	0.1974	-0.119	0.3668	0.085723	0.0436	0.135	0.0012	-1.128	0.1064	-0.711	0.3055
Normalized Walking Speed												
Mean (%)	80	0.2279	0.000121	0.9996	-0.126	0.0897	-0.294	<.0001	0.580	0.6344	1.382	0.2578
CV (%)	80	0.1974	-0.119	0.3668	0.085723	0.0436	0.135	0.0012	-1.128	0.1064	-0.711	0.3055

Abbreviations: PTA, pure tone average; DHI-S, Dizziness Handicap Inventory – Screening version score; nhwhite, non-Hispanic white; CV, coefficient of variation; R^2, coefficient of determination; B, unstandardized regression coefficient
